# *Spodoptera frugiperda* X-Tox Protein, an Immune Related Defensin Rosary, Has Lost the Function of Ancestral Defensins

**DOI:** 10.1371/journal.pone.0006795

**Published:** 2009-08-27

**Authors:** Delphine Destoumieux-Garzón, Michel Brehelin, Philippe Bulet, Yvan Boublik, Pierre-Alain Girard, Stephen Baghdiguian, Robert Zumbihl, Jean-Michel Escoubas

**Affiliations:** 1 CNRS, UMR5119 Laboratoire Ecosystèmes Lagunaires, CC80, Montpellier, France; 2 IFREMER, UMR 5119 Laboratoire Ecosystèmes Lagunaires, CC80, Montpellier, France; 3 Université Montpellier 2, UMR5119 Laboratoire Ecosystèmes Lagunaires, CC80, Montpellier, France; 4 INRA, UMR 1133 Laboratoire Écologie Microbienne des Insectes et Interactions Hôte-Pathogène (EMIP), CC54, Montpellier, France; 5 Université Montpellier 2, UMR1133 Laboratoire EMIP, CC54, Montpellier, France; 6 Université Joseph Fourier, CNRS, UMR5525, TIMC-IMAG, Bat Le Forum Plateforme BioPark d'Archamps, Archamps, France; 7 CNRS, UMR5237 Plateforme Protéines recombinantes, Montpellier, France; 8 Université Montpellier 2, CNRS, Institut des sciences de l'évolution UMR5554, CC63, Montpellier, France; CNRS/Université de Toulouse, France

## Abstract

**Background:**

X-tox proteins are a family of immune-related proteins only found in Lepidoptera and characterized by imperfectly conserved tandem repeats of several defensin-like motifs. Previous phylogenetic analysis of X-tox genes supported the hypothesis that X-tox have evolved from defensins in a lineage-specific gene evolution restricted to Lepidoptera. In this paper, we performed a protein study in which we asked whether X-tox proteins have conserved the antimicrobial functions of their ancestral defensins and have evolved as defensin reservoirs.

**Methodology/Principal Findings:**

We followed the outcome of Spod-11-tox, an X-tox protein characterized in *Spodoptera frugiperda*, in bacteria-challenged larvae using both immunochemistry and antimicrobial assays. Three hours post infection, the Spod-11-tox protein was expressed in 80% of the two main classes of circulating hemocytes (granulocytes and plasmatocytes). Located in secretory granules of hemocytes, Spod-11-tox was never observed in contact with microorganisms entrapped within phagolyzosomes showing that Spod-11-tox is not involved in intracellular pathogen killing. In fact, the Spod-11-tox protein was found to be secreted into the hemolymph of experimentally challenged larvae. In order to determine antimicrobial properties of the Spod-11-tox protein, it was consequently fractionated according to a protocol frequently used for antimicrobial peptide purification. Over the course of purification, the anti-Spod-11-tox immunoreactivity was found to be dissociated from the antimicrobial activity. This indicates that Spod-11-tox is not processed into bioactive defensins in response to a microbial challenge.

**Conclusions/Significance:**

Altogether, our results show that X-tox proteins have not evolved as defensin reservoirs and have lost the antimicrobial properties of the ancestral insect defensins. The lepidopteran X-tox protein family will provide a valuable and tractable model to improve our knowledge on the molecular evolution of defensins, a class of innate immune effectors largely distributed over the three eukaryotic kingdoms.

## Introduction

Innate immunity is the sole line of defense of invertebrates. Their immune response relies on diverse humoral and cellular activities taking place both at local and systemic levels. The invertebrate immune response has been extensively studied in insects and today it is in the insect model, *Drosophila melanogaster*, that we have the most integrated understanding of this physiological function. Indeed, biochemical, genetic and molecular biology approaches have led to the characterization of the molecular mechanisms involved in (i) pathogen recognition, (ii) signal transduction through extra- and intra-cellular signaling pathways, and (iii) pathogen elimination through the production of effectors molecules and cell activation (for review see [1], [2]). Completion of genomic sequences from members of the four major insect orders and subsequent comparative analysis indicate that the basic set of molecules defining the *D. melanogaster* immune repertoire is conserved across Diptera, Coleoptera, Hymenoptera, and Lepidoptera [3]–[6].

Albeit the immune system framework seems to be conserved across insects, specific characteristics are observed in some insect orders. Thus, hemolin is a bacteria-inducible pattern recognition protein of the immunoglobulin superfamily, which is specific of the Lepidoptera immune system [7], [8], [9]. Recently, we have also discovered a new family of immune-related genes, restricted to Lepidoptera, which encode the so-called X-tox proteins [10]. This is a unique family of genes, whose deduced amino-acid sequence is composed of a variable number (X) of cysteine-stabilized alpha beta motifs (CS-αβ). This motif is characteristic of invertebrate defensins and scorpion toxins [11]. In the *Spodoptera frugiperda* (Lepidoptera, Noctuoidea) Spod-11-tox protein, 11 cationic domains with a CS-αβ motif share the following consensus sequence: C-x_4_-C-x_3_-C-x_7_-G-x-C-x_3_-K/R-C-x-C. Similarly, there are six CS-αβ motifs in *Galleria mellonella* (Lepidoptera, Pyraloidea) [12] and five to six in *Bombyx mori* (Lepidoptera, Bombycoidea) [13]. Phylogenetic analysis supports the hypothesis that X-tox proteins, which are evolutionary derived from lepidopteran defensins, represent a new family of proteins restricted to Lepidoptera. In a previous study, *S. frugiperda spod-11-tox* gene expression was shown to be rapidly induced upon experimental infection, and unlike insect defensin genes, blood cells were identified as the main site of *spod-11-tox* gene expression.

Puzzled by the combination of 11 variants of defensin-like peptides in a single protein, we asked whether Spod-11-tox is a reservoir of defensins with antimicrobial activities. To answer that question, we performed a protein study in which we monitored the outcome of the Spod-11-tox protein in terms of tissue localization and putative processing, in insects exposed to a microbial challenge. We first raised polyclonal antibodies directed against rSpod-11-tox and showed that during a bacterial infection, Spod-11-tox rapidly accumulates within secretory granules of the two main classes of hemocytes (granulocytes and plasmatocytes) in a membrane-associated form. Spod-11-tox expression was found to be independent of the phagocytic activity of hemocytes and the protein never co-localized with phagocytosed microorganisms, showing that the Spod-11-tox protein is not involved in intracellular pathogen killing and probably not in non-self-recognition neither. Because Spod-11-tox was found to be rapidly secreted into the hemolymph following challenge, it may play a role in the systemic immune response. In the insect plasma (cell-free hemolymph), the anti-Spod-11-tox immunoreactivity was dissociated from the antimicrobial activities, as determined following purification in conditions known to preserve antimicrobial properties. Altogether, our results show that although Spod-11-tox is organized in a cluster of 11 defensin motifs, this large protein is not a reservoir of what is referred as antimicrobial defensins.

## Materials and Methods

### Insects and Immune Challenge

*S. frugiperda* was reared on artificial diet at 23°C with a photoperiod of 12 h. Sixth-instar larvae were used for the expression studies. Experimental infections were performed by an injection of 20 µL of PBS-washed microorganisms per larvae (numbers of microorganisms injected are indicate in figure captions). Two bacterial strains were used, namely *Escherichia coli* CIP7624 (Gram-negative) and *Micrococcus luteus* CIP5345 (Gram-positive), as well as the yeast strain *Pichia pastoris* (GS115 from Invirogen^TM^).

### Raising Specific Antibodies to Spod-11-tox

A New-Zealand rabbit was first subcutaneously injected with an emulsion of 80 µg of the purified rSpod-11-tox solubilized in complete Freund's adjuvant (CFA; Gibco-BRL), and then intramuscularly boosted twice with 80 µg of rSpod-11-tox solubilized in incomplete Freund's adjuvant (IFA; Gibco-BRL). Finally, rabbit was intramuscularly boosted four times at 1-month intervals with 80 µg of rSpod-11-tox solubilized in IFA. The rabbit was killed and the whole serum was collected. Polyclonal antibodies to rSpod-11-tox were affinity-purified through a column of rSpod-11-tox coupled to NHS-activated HP matrix (GE Healthcare), according to the protocol recommended by the manufacturer. All experiments using rabbits were conducted in the DSV-accredited animal facility at the Molecular Biochemistry Research Center (agreement number: C3417204) and handled in accordance with institutional guidelines. All experiments using rabbits were approved by the Ethics Committee of the University of Montpellier 2.

### Protein extracts and Western blotting

Hemolymph was collected, from a cut abdominal proleg, into ice-cold anti-coagulant buffer (69 mM KCl, 27 mM NaCl, 2 mM NaHCO_3_, 100 mM D-glucose, 30 mM tripotassium citrate, 26 mM citric acid, 10 mM Na_2_–EDTA, pH 4.6, 420 mOsm). Hemocytes and plasma were separated by centrifugation at 600×g during 30 seconds at 4°C. Hemocytes were washed once in ice-cold PBS and once in buffer A (20 mM Tris-HCl, pH 7.5, 3 mM MgCl_2_, 1 mM EDTA, containing the complete protease inhibitor cocktail from Roche at a concentration of one tablet for 10 mL). Hemocyte lysis was performed using buffer A in the presence of 0.1% Triton X-100. The membrane and cytosolic fractions were prepared in buffer A using a Dounce homogenizer and centrifugation at 14,000×g for 30 min at 4°C. The cytosolic fraction was removed, and the crude membrane fraction was washed once in buffer A and dissolved in the same buffer. The protein content was measured by the Bradford assay from Bio-Rad using Bovine Serum Albumin (BSA) as standard. Proteins were separated by SDS-PAGE under reducing conditions, and transferred onto membranes (Immun-Blot^TM^ PVDF 0,22 µm membrane from Bio-Rad). Membranes were probed with polyclonal anti-Spod-11-tox antibodies diluted at 120 ng/mL in PBS; 0.05% Tween-20; 1% BSA. A horseradish peroxidase-coupled anti-rabbit IgG (GE Healthcare) was used at a 1∶5,000 dilution for detection by chemiluminescence (ECL Western blotting detection, GE Healthcare) and was then exposed to Hyperfilm-ECL (GE Healthcare).

### Immunolabelling and Microscopy

Hemocyte monolayers were prepared as previously described [14]. Briefly, the collected hemocytes were resuspended in 1 mL of PBS. Hemocyte suspensions were layered on 12 mm diameter heat-sterilized glass coverslips in 24 well plates, and cells were allowed to adhere on glass for 10 min at room temperature. Cells were fixed in 3.7% formaldehyde, 0.25% glutaraldehyde in PBS for 20 min, washed with PBS, incubated in 50 mM NH_4_Cl for 10 min and washed with PBS plus 0.2% BSA. Cells were permeabilized in 0.4% Triton X-100 in PBS for 4 min, rinsed in PBS and incubated with rabbit anti-Spod-11-tox antibody (1.2 µg/mL) at room temperature for 20 min. After washing in PBS, samples were incubated at room temperature for 20 min with TRITC (Tetra methyl Rhodamine Iso Thio Cyanate)-conjugated goat anti-rabbit secondary antibody (Sigma T6778; dilution 1∶400) and observed with a Leica TCS 4D Laser Confocal Microscope. Processing of cells in the absence of primary antibody served as a negative control. DNA was visualized with DAPI (Di Aminido Phenyl Indol). The constitutive green fluorescent protein (GFP)-labelled *E. coli* XL1 (DE3) was a generous gift from A. Givaudan [15].

For immunogold labelling in TEM, hemocytes or insect tissues were fixed in 3.7% formaldehyde plus 0.25% glutaraldehyde in PBS for 2 h, dehydrated in alcohol and embedded in Unicryl^TM^ resin according to the manufacturer. Ultrathin sections were incubated in rabbit anti-Spod-11-tox antibody at room temperature for 2 h, rinsed and incubated in immunogold labelled protein A, then finally counterstained with 2.5% uranyl acetate for 5 min.

### Plasma Purification Procedure

#### Extraction and prepurification by solid-phase extraction (SPE)

Plasma from PBS-challenged (5.2 mL) or live bacteria-challenged (28 mL) larvae, was acidified to pH 2 with 1 M HCl. The acidic extraction was performed over night under gentle shaking at 4°C. After centrifugation (16,000 *g* for 30 min at 4°C), the supernatant was prepurified by SPE on Sep-Pak C_18_ cartridges (Waters) equilibrated with acidified water (0.05% trifluoroacetic acid, TFA). Elutions were performed with 10, 40, and 80% acetonitrile in acidified water. All fractions were freeze-dried in a vacuum centrifuge (Speed-Vac, Cryo Rivoire) and subsequently reconstituted with MilliQ water at 1/20 of the initial hemolymph volume.

#### HPLC purification

*STEP 1 -* The 40% Sep-Pak fractions were subjected to reversed-phase chromatography on an UP5NEC25QS column (Interchim) equilibrated in acidified water. Separation of the 40% Sep-Pak fractions was performed with a linear gradient of 0-60% acetonitrile in acidified water over 80 min at a flow rate of 1 mL/min.

*STEP 2 -* Different gradients were used for this second purification step. Immunoreactive fractions were purified on the same reversed-phase column as in STEP 1 at a controlled temperature of 35°C. Linear biphasic gradients were used according to the following calculation, for a fraction eluted with X% acteonitrile, the gradient was composed of 0-(X-6)% acetonitrile in acidified water over 5 min, and of (X−6)%-(X+9)% over 45 min at a flow rate of 1 mL/min.

*STEP 3 –* When needed, the fractions of interest were subjected to a final purification step on a narrow-bore reversed-phase column (Xbridge BEH130, Waters Associates) at 40°C at a flow rate of 0.25 mL/min using the biphasic gradients described in STEP 2.

All HPLC purifications were performed on a Waters HPLC system (Waters 600 pump) equipped with a photodiode array (Waters 996 PDA). Column effluent was monitored by its UV absorption at 225 nm. Fractions corresponding to absorbance peaks were hand collected in polypropylene tubes (Microsorb 75 mm×12 mm, Nunc immunotubes), concentrated under vacuum (Savant) and reconstituted in MilliQ water (Millipore^TM^) before antimicrobial activity and immunoreactivity testing.

### Antimicrobial Assays

Antimicrobial activity was assayed against two bacteria, *Micrococcus luteus* (CIP5345), and *Escherichia coli* (SBS 363 or CIP7624), and against the filamentous fungus *Fusarium oxysporum* (generous gift from A. Vey, INRA Saint Christol-lès-Alès) based on the liquid growth inhibition assay described by Hetru and Bulet [16]. Poor Broth (PB: 1% bacto-Tryptone, 0.5% NaCl w/v, pH 7.5), and ½ Potato Dextrose Broth (Difco) were used for bacterial and fungal growth, respectively. Growth of bacteria was monitored spectrophotometrically at 600 nm on a multifunctional microplate reader (Tecan infinite 200) while fungal growth was evaluated after 24 and 48 hours at 30°C by optical microscopy and measurement of the culture absorbance at 595 nm.

### Dot-blot ELISA assay

Anti-Spod-11-tox immunoreactivity of HPLC fractions was measured by spotting 1 µL of each fraction on membranes (Immun-Blot^TM^ PVDF 0,22 µm membrane from Bio-Rad). Membranes were probed with anti Spod-11-tox antibodies (as described in “Protein extracts and Western blotting”).

## Results

### Expression pattern of Spod-11-tox during immune response

**Figure 1:**
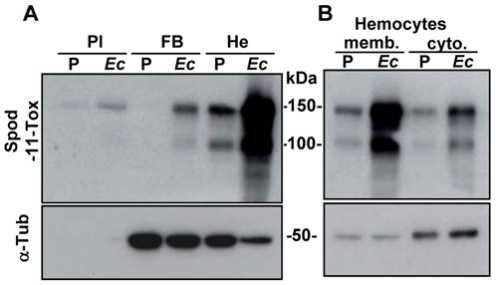
Expression pattern of Spod-11-tox during the immune response. A) Spod-11-tox is mainly expressed in hemocytes upon immune challenge. Western blot analyses were performed with protein extracts from plasma (Pl), fat body (FB) and hemocytes (He) of *S. frugiperda* larvae 24 h post injection of PBS (P) or *E. coli* (*Ec*, 10^5^ bacteria/larvae). Proteins were loaded (20 µg/lane) on a 10% SDS-PAGE. The blot was successively probed with both a polyclonal anti-Spod-11-tox antibody (Spod-11-tox, upper panel) and an anti-α-tubulin antibody (α-Tub, lower panel). The absence of a signal with α-tubulin antibody indicates that the plasma preparations are not contaminated by cells. B) Sub cellular location of Spod-11-tox. Membrane (memb.) and cytosolic (cyto.) fractions were prepared with hemocytes withdrawn from *S. frugiperda* larvae (challenged as described in A) and separated on a 10% SDS-PAGE. Protein separation and western blot were performed as in A.

To investigate the outcome of the Spod-11-tox protein during the immune response we produced antibodies directed against recombinant protein (rSpod-11-tox). These antibodies were used in western blot experiments to monitor the expression of the Spod-11-tox protein in larvae subjected to PBS or *E. coli* injections. In agreement with our previous gene expression data [10], Spod-11-tox was mainly produced by hemocytes of bacteria-challenged insects (Figure 1A). Two immunoreactive bands were detected by anti-Spod-11-tox antibodies that likely correspond to the products of the two transcripts previously revealed by Northern Blot analysis [10].

In order to define more precisely the cellular location of Spod-11-tox, we prepared membrane and cytosolic fractions from *S. frugiperda* larvae hemocytes. Western blots showed that Spod-11-tox is mainly recovered into the membrane fraction suggesting that the protein is associated to cell membranes or to intracellular organelles (Figure 1B).

Altogether, these results indicate that in response to a bacterial infection, Spod-11-tox rapidly accumulates into hemocytes, either bound to cell membranes or stored in intracellular organelles. Moreover, Spod-11-tox was also observed in plasma. Since no trace of α-tubulin was found in the plasma, the presence of Spod-11-tox in plasma is due to secretion of the protein rather than cell lysis.

### Hemocyte cell types involved in Spod-11-tox production

**Figure 2:**
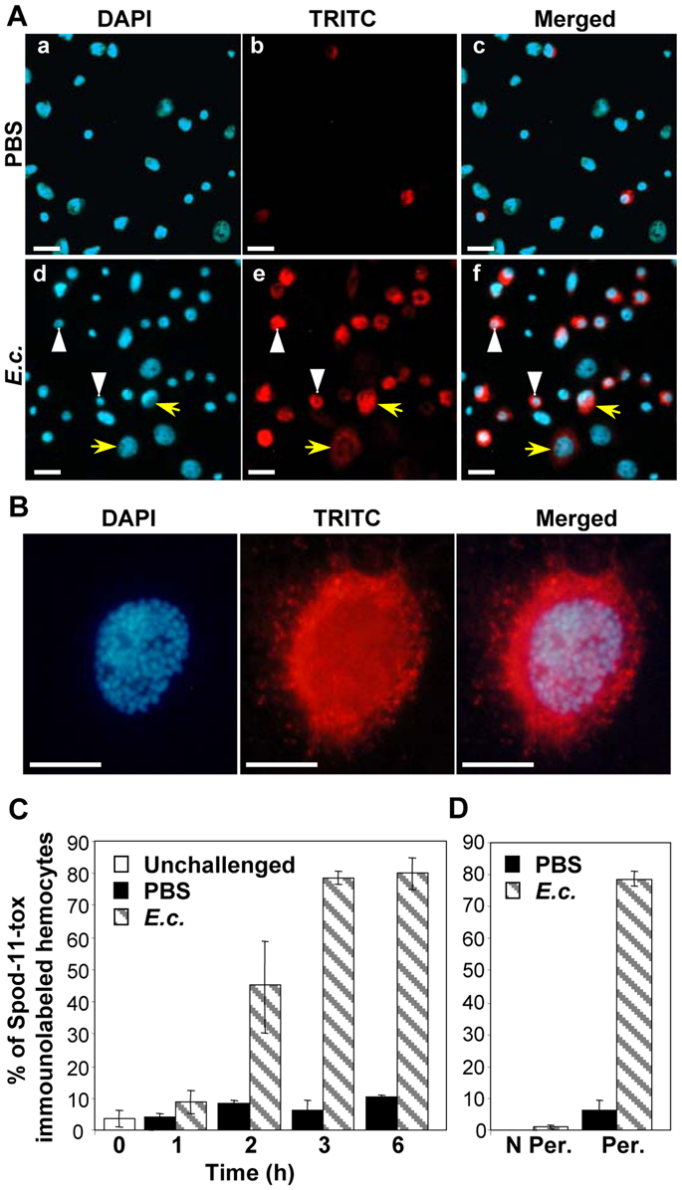
Hemocyte cell types involved in Spod-11-tox production. A) Immunofluorescence staining of Spod-11-tox in hemocytes withdrawn from *S. frugiperda* larvae 6 h post injection with PBS (a, b, c) or *E. coli* (10^5^ bacteria/larvae) (d, e, f). Cells were DAPI stained (a, d) and immunolabelled with rabbit anti-Spod-11-tox revealed by TRITC-conjugate anti-rabbit (b, e). Overlays of DAPI and TRITC were shown in c and f. Yellow arrows and white arrowheads indicate plasmatocytes and granulocytes respectively. Bars = 20 µm. B) Spod-11-tox appeared mainly aggregated within the cytoplasm in a manner evocative of granule-like vesicles. Higher magnification of a plasmatocyte withdrawn from *S. frugiperda* larvae 6 h post injection of *E. coli* (10^5^ bacteria/larvae). Bars = 10 µm. C) Relative percentage of Spod-11-tox immunolabelled circulating hemocytes. Same experiment as in A except that hemocytes were withdrawn at the indicated times. Data are presented as mean±SD of at least three independent experiments (a minimum of 2,000 hemocytes/condition were counted). D) Intracellular location of Spod-11-tox. Same experiment as in B except that hemocytes were withdrawn 3 h post challenge and were (Per.) or were not (N Per.) permeabilized before immunolabelling.

To define the hemocyte cell types involved in Spod-11-tox production, we performed immunofluorescence on hemocytes collected from *S. frugiperda* last instars larvae 6 h post challenge. In PBS-treated larvae, only few hemocytes were immunoreactive to anti-Spod-11-tox antibodies whereas in *E. coli*-treated larvae, most hemocytes were immunolabelled (Figure 2A, b and e). Plasmatocytes and granulocytes, which represent approximately 85–95% of all hemocytes in last instars larvae [17], are easily identified according to their nuclei size (Figure 2A, d; plasmatocytes, yellow arrow; granulocytes, white arrowhead). Both cell types were recognized by the anti-Spod-11-tox antibodies (Figure 2A, e and f). At higher magnification, Spod-11-tox appeared mainly aggregated within the cytoplasm in a manner evocative of granule-like vesicles (Figure 2B).

Interestingly, 80% of the cells were labelled as soon as 3 h after septic injury, while the percentage of Spod-11-tox-labelled hemocytes remained low in PBS-treated and not significantly different from unchallenged larvae (Figure 2C). The total lack of labelling of non-permeabilized hemocytes (Figure 2D) demonstrated the intracellular location of the protein.

Altogether, these results demonstrate that, *in vivo*, a bacterial-challenge rapidly induces Spod-11-tox synthesis and accumulation in granulocytes and plasmatocytes, the two main classes of hemocytes.

### Subcellular localization of Spod-11-tox during phagocytosis

**Figure 3:**
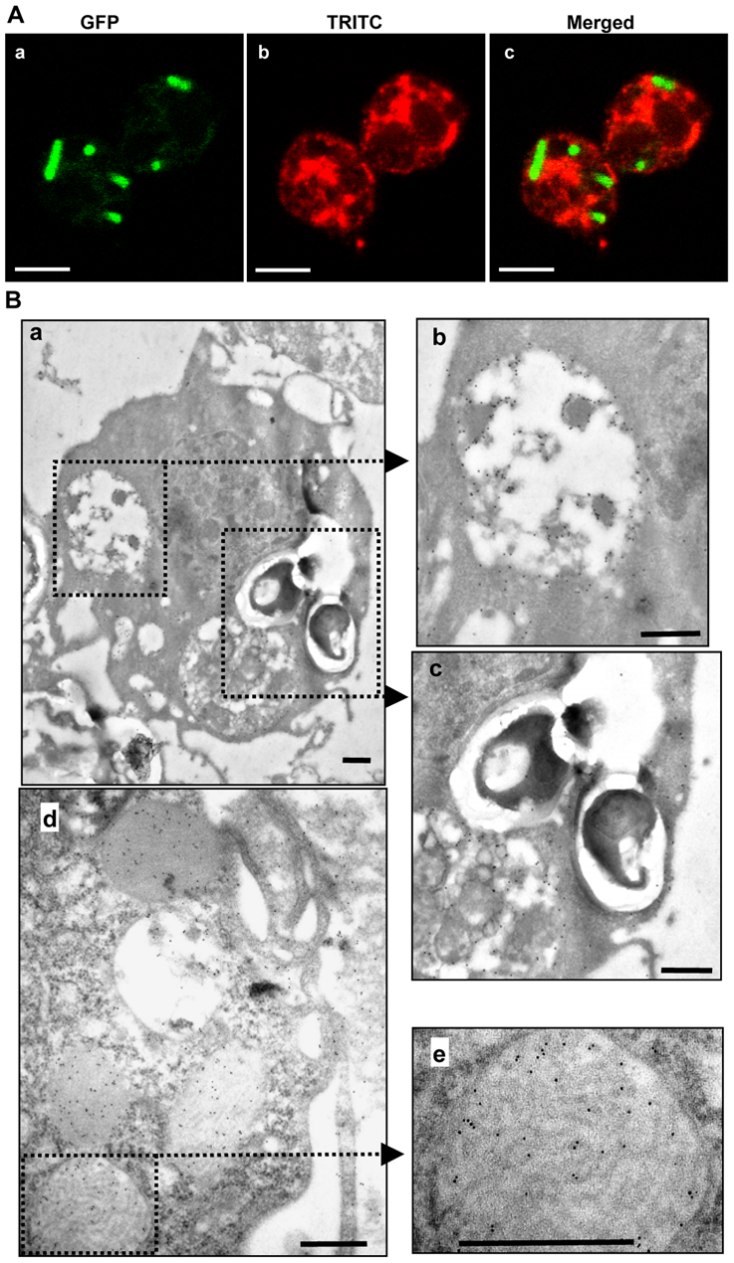
Subcellular localization of Spod-11-tox. A) Confocal microscopy of phagocytosed *E. coli*. Immunofluorescence staining of Spod-11-tox containing hemocytes withdrawn from *S. frugiperda* larvae 3 h post injection of GFP-expressing *E. coli* (10^7^ bacteria/larvae). Cells were immunolabelled with rabbit anti-Spod-11-tox revealed by TRITC-conjugate anti-rabbit. Bars = 5 µm. B) Immunoelectron microscopy of granulocytes withdrawn from *S. frugiperda* larvae 3 h post injection of *Pichia pastoris* (10^6^ yeast/larvae). Note the presence of heterogeneous bodies looking like secondary lysosomes (b), phagocytosed *P. pastoris* (c) and structured granules (e). Immunogold labelling using 10 nm colloidal gold-conjugated Protein A. Bars = 0.5 µm.

The intracellular location of Spod-11-tox in granule-like vesicles raised the question of its involvement in intracellular bacterial killing. We therefore examined whether Spod-11-tox-expressing hemocytes were preferentially involved in bacterial phagocytosis using a GFP-expressing *E. coli* strain. Phagocytosed bacteria were observed in both Spod-11-tox-labelled and unlabelled hemocytes. In addition, immunolabelled cells free of phagocytosed bacteria were also observed, suggesting that Spod-11-tox expression was not associated to phagocytosis (data not shown). Moreover, confocal microscopy showed that bacteria are found in a cell compartment devoid of Spod-11-tox (Figure 3A). Similar results were obtained when hemocytes withdrawn from *S. frugiperda* larvae 3 h after an injection of *Pichia pastoris* were immunogold-labelled with anti-Spod-11-tox antibodies. Indeed, gold particles revealing the presence of Spod-11-tox were not present in yeast-containing phagocytic vacuoles (Figure 3B, a and c). Moreover, immunogold electron microscopy further localized Spod-11-tox in hemocyte heterogeneous bodies (Figure 3B b) and in structured granules (Figure 3B, d and e). The later organelles are typical inclusions of granular cells in most insect orders [18] and are released into the plasma upon infection [19].

These results showed that Spod-11-tox expression is independent of hemocyte phagocytosis and that the protein does not co-localize with phagocytosed microorganisms suggesting that the protein is not involved in intracellular pathogen killing. In addition, the absence of co-localization suggests that Spod-11-tox is not involved in non-self-recognition. This is supported by immunolabelling experiments showing that, *in vitro*, rSpod-11-tox does not directly interact with *E. coli* or *P. pastoris* (data not shown).

### Spod-11-tox is secreted into the hemolymph but not processed into bioactive defensins

**Figure 4:**
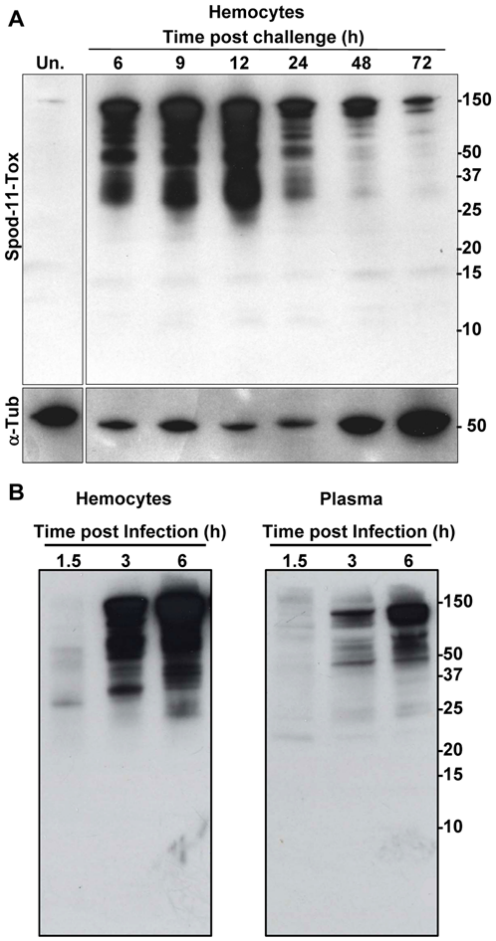
Time course analysis of Spod-11-tox expression in challenged *S. frugiperda* larvae (*E. coli* 10^5^ bacteria/larvae). A) Western blot analyses performed with protein extracts from hemocytes (from 6 to 72 hours post infection). B) Comparison of Spod-11-tox content in hemocytes and plasma early post infection. Proteins were loaded (10 µg/lane) on a Tris-Tricine 10–20% gradient gel. The western blot was realized as in figure 1. Numbers on the right sides of the blots indicate the molecular weight (kDa) estimated using Precision Plus Protein^TM^ Standards from Bio-Rad. Un; unchallenged.

The intracellular localization of Spod-11-tox suggested a secretion of Spod-11-tox proteins into the insect plasma. We therefore performed a time-course analysis of Spod-11-tox expression and secretion. Insect hemolymph was collected at different time intervals post-microbial challenge and both plasma and hemocytes were subjected to western-blot analysis for Spod-11-tox detection. Spod-11-tox was almost undetectable in protein extracts of hemocytes from unchallenged larvae, the protein massively accumulated in hemocytes until 12 h post-infection and then decreased (Figure 4A). A similar profile was obtained with plasma except that Spod-11-tox detection was delayed compared to hemocytes extracts (Figure 4B).

The Spod-11-tox protein that we found here to be secreted into the insect hemolymph upon microbial challenge was also found to be subjected to major processing. Indeed, a succession of bands (around 10) spreading out over the 25–150 kDa range was observed in extracts prepared from hemocytes collected 6–12 h post infection. This indicates that Spod-11-tox, with a calculated molecular mass at 75.9 kDa, is naturally processed in smaller forms. In the Tris-Tricine 10–20% gradient gel used, no immunoreactive band was observed below 10 kDa, i.e. the molecular mass range where defensin-like domains may be expected, neither in hemocytes nor in plasma. To ensure that putative low molecular mass molecules can be recognized, recombinant Spod-11-tox was cleaved by the V8 protease and analyzed by western blot. Under these conditions, anti-Spod-11-tox antibodies were able to detect peptides as small as 3 kDa, which nonetheless were devoid of antimicrobial activity (data not shown). These results strongly suggest that the cleavage or maturation of Spod-11-tox observed in hemocytes during the first 24 hours of infection does not generate defensin-like subunits.

**Figure 5:**
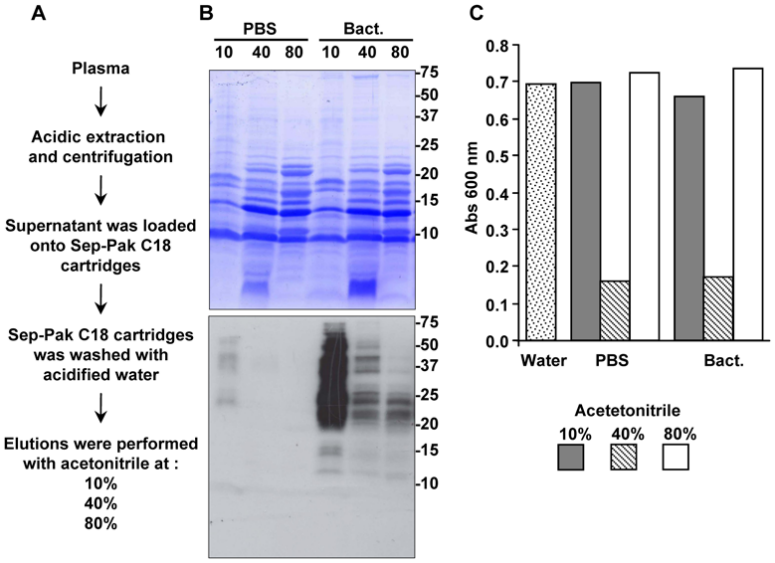
Purification procedure and properties of *S. frugiperda* hemolymph. A) Schematic representation of the protocol used for the purification of the insect hemolymph. Proteins were eluted from the Sep-Pack C_18_ cartridge with 10, 40, and 80% acétonitrile prepared in acidified water (TFA 0.05%), lyophilizated and reconstituted with MilliQ water. B) Western blot analysis of Spod-11-tox content in the elution fractions of Sep-Pack C_18_ cartridge. Protein extracts were loaded (15 µg/lane) on a Tris-Tricine 16.5% gel and blotted on a PVDF membrane. The blot was Coomassie stained (upper panel) and probed with polyclonal anti-Spod-11-tox antibody (lower panel). Numbers on the right sides of the blots indicate the molecular weight (kDa) estimated using Precision Plus Protein^TM^ Standards from Bio-Rad. C) The eluted Sep-Pack fractions were tested for their antimicrobial activities by liquid growth inhibition assay against *E. coli* (CIP7624). Results are representative of three independents experiments.

To determine if the Spod-11-tox secreted into the insect hemolymph over the immune response is matured into defensin peptides, *S. frugiperda* plasma was fractionated according to a protocol widely used to isolate and characterize antimicrobial peptide [20]. Five hundred sixth instar larvae were challenged by injection of bacteria, and the hemolymph (28 mL final volume) was collected after 8 h. In a parallel experiment, hemolymph from PBS-challenged larvae was treated under the same conditions. The plasma of insects was then acidified and fractionated by solid phase extraction on reversed phase Sep-Pak cartridges (Figure 5A).

While anti-Spod-11-tox immunoreactivity was specifically found in the Sep-Pak fractions obtained from bacteria-challenged larvae (Figure 5B), similar profiles of antimicrobial activity were found for PBS- and bacteria-challenged larvae (Figure 5C). More specifically, the 10% Sep-Pak fraction from bacteria-challenged larvae, which contained most of the anti-Spod-11-tox immunoreactivity (Figure 5B), had no activity against the micro-organism tested. In fact, most of the antimicrobial activity was found in the 40% Sep-Pack fraction (Figure 5C). Conversely, no immunoreactivity against Spod-11-tox was found in the 40% fraction from PBS-injected larvae (Figure 5B), whereas this fraction was clearly active against *E. coli* (Figure 5C).

**Figure 6:**
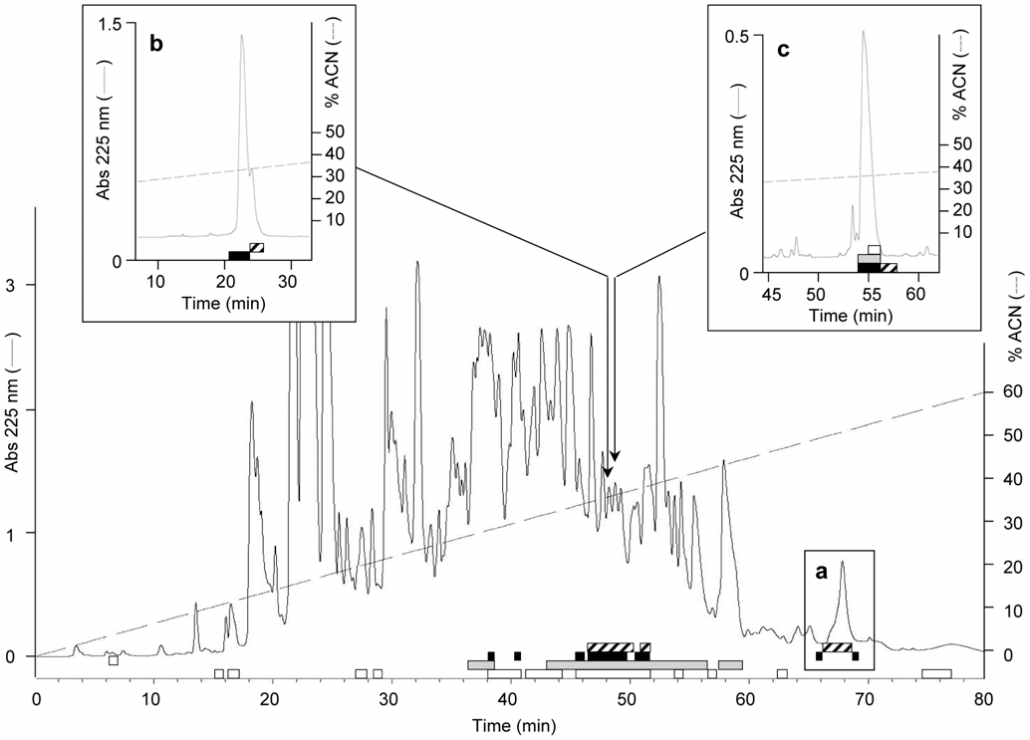
Discrimination between antimicrobial properties and anti-Spod-11-tox immunoreactivity of the fractionated *S. frugiperda* hemolymph. The 40% Sep-Pack fraction from the extraction of immunized *S. frugiperda* plasma was subjected to reversed-phase HPLC on an Interchim UPNEC25QS column using a 0–60% linear gradient (dashed line) of acidified acetonitrile over 80 min. Antimicrobial activity against *E. coli* SBS363 (black rectangles) *M. luteus* (grey rectangles) and *F. oxysporum* (white rectangles) was measured by liquid growth inhibition assays. Anti-Spod-11-tox immunoreactivity (hatched rectangles) was measured by dot-blot ELISA. Insets show the dissociation between immunoreactive and antimicrobial fractions over the course of the purification. a: at the first purification step; b: at the second purification step (same column with a 0-27-42% biphasic linear gradient of acidified acetonitrile over 5 and 40 min); c: at the third purification step (Xbridge BEH300 narrowbore column with a 0-23-43% biphasic gradient of acidified acetonitrile over 5 and 80 min). Absorbance (Abs) was monitored at 225 nm.

Further purification of the plasma collected from bacteria-challenged larvae revealed the absence of Spod-11-tox-derived defensins in the 40% Sep-Pak fraction, which is reported to contain, in the *Heliothis virescens* model at least the heliomicin, a lepidopteran defensin [20]. Indeed, no anti-Spod-11-tox immunoreactive peptide was observed below 10 kDa on SDS-PAGE of the Sep-Pak-fractionated plasma (Figure 5B), while defensin molecular masses are expected at ∼4 kDa. In addition, when separated by reversed-phase HPLC using a protocol adapted for defensin isolation, the 40% Sep-Pak-fractionated plasma from bacteria-challenged larvae displayed dissociated antimicrobial activity and anti-Spod-11-tox immunoreactivity. This was demonstrated by subjecting all HPLC-collected fractions to antimicrobial assays against *E. coli* (Gram-negative bacterium), *M. luteus* (Gram-positive bacterium), and *Fusarium oxysporum* (filamentous fungus) on the one hand, and to an anti-Spod-11-tox dot-blot ELISA on the other hand. Thus, after a first purification step, some immunoreactive fractions (eluted around 52% acetonitrile) already lacked antimicrobial activity (Figure 6, inset a). Fractions which displayed both antimicrobial activity and immunoreactivity (eluted around 35% acetonitrile, Figure 6), where further purified with the objective of identifying Spod-11-tox-derived defensins. However, in all cases, dissociation between the immunoreactivity and the antimicrobial activity was observed over the course of the purification, as shown for two of them in Figure 6 (inset b and c). Altogether, our data strongly suggest that Spod-11-tox is not processed into defensins, which in Lepidoptera, are reported to be antifungal.

## Discussion

The data presented in this study of the Spod-11-tox protein showed that this acute phase protein is produced upon microbial infection by granulocytes and plasmatocytes (the two main classes of circulating hemocytes) and processed into lower molecular masse components before being rapidly secreted into the plasma where it participates to a systemic response rather than being involved in intracellular pathogen killing.

In response to infection, Spod-11-tox was found to accumulate in heterogeneous bodies that are present in both plasmatocytes and granulocytes and in structured granules, (granulocyte-specific organelles) involved the protein secretion into the plasma. This later localization of Spod-11-tox, in addition to the presence of a signal peptide on the amino-terminal part of the protein, supports the involvement of Spod-11-tox in the systemic immune response. Indeed, Spod-11-tox expression in hemocytes is independent of phagocytosis and Spod-11-tox does not co-localize with phagocytosed microorganisms. This is in contrast with mammalian defensins which are stored in leukocyte granules that fuse to phagocytic vacuoles and deliver defensins onto the target microorganism [21]. Storage of immune proteins including antimicrobial peptides (AMPs) in hemocyte granules has already been reported in invertebrates. For example, AMPs are constitutively present in hemocytes of the mussel *Mytilus galloprovincialis* [22], the shrimp *Penaeus vannamei* [23], the horseshoe crab *Tachypleus tridentatus* [24], and the termite *Pseudacanthotermes spiniger* [25]. However, in those organisms, AMPs are constitutively present and not induced upon a microbial challenge as observed for Spod-11-tox.

Intense processing of Spod-11-tox was observed within hemocytes of challenged insects, which is then released into the blood stream. However, we showed here that the insect plasma, which is rich in antimicrobials, is devoided of bioactive Spod-11-tox-derived defensins. In agreement, in the present study, *in vitro* cleavage of rSpod-11-tox with V8 protease failed to generate peptides with antimicrobial activity. The lack of organized processing as found in other AMP precursors [26], [27] is also supported by previous sequence analysis of Spod-11-tox, which showed that the spacer region bridging the Spod-11-tox CS-αβ motifs are not conserved and lack potential conserved cleavage sites that could be recognized by a specific processing enzyme [10].

Altogether, the data obtained in this work indicate that Spod-11-tox, whose structure is evocative of a defensin rosary, is not processed into bioactive defensin peptides. This rules out the hypothesis of Spod-11-tox being a precursor for defensins, whose sequence diversity may result in complementary and/or synergistic activities beneficial for insect defense. This conclusion is corroborated by studies conducted on two other lepidopteran species. In *Heliothis virescens*, an EST program performed on immune-stimulated hemocytes lead to the characterization of an *x-tox* gene [28]. The amino acid sequence deduced from this gene revealed that it contains three CS-αβ motifs (GenBank accession number ACI02333). Interestingly, none of them correspond to antimicrobial peptides, including heliomicin the *H. virescens* defensin, purified from this insect in our previous systematic analysis of *H. virescens* plasma [20]. In *G. mellonella*, three defensins were purified by biochemical approaches similar to the one used in this work [29], [30] and none of them derived from the proteolytic cleavage of Gall-6-tox (the X-tox protein characterized in *G. mellonella* [12]). This suggests that in *H. virescens* and in *G. mellonella*, like in *S. frugiperda*, X-tox proteins are not processed into individual bioactive defensins.

Because the antimicrobial activity of insect plasma was found not to be associated to the presence of Spod-11-tox-derived defensins nor to the presence of larger Spod-11-tox processing products, we believe that overall, Spod-11-tox is not directly involved in the microorganism killing. Expression studies revealed that in *S. frugiperda* three defensin genes (*spodoptericin*, *Sf*-*gallerimycin* and *Sf*-*cobatoxin*) are up regulated, to a variable degree, upon challenge [31]. Thus, during immune response, *S. frugiperda* produces simultaneously defensins and Spod-11-tox protein. Similar results were obtained for *G. mellonella* [12], [30]. Altogether, these observations lead us to hypothesize that X-tox proteins evolved to a distinct function. This function is most likely unrelated to pathogen recognition and opsonisation since Spod-11-tox is not expressed at hemocyte surface, does not interact with pathogens, and does not co-localize with phagocytosed microorganisms.

In conclusion, we showed here that, through evolution, Spod-11-tox and probably all X-tox proteins, have lost the function of ancestral insect defensins. To gain more insight into the immune function(s) of X-tox proteins, other experimental approaches, such as gene knock down will be needed. What driving force allowed the evolution of defensins in X-tox proteins in Lepidoptera solely remains to be determined. We believe that through the X-tox protein family, Lepidoptera will provide a valuable and tractable model to improve our knowledge on the molecular evolution of defensins, a class of innate immunity effectors largely distributed over the three eukaryotic kingdoms.
